# Effect of WeChat-Based Education on the Quality of Intestinal Preparation Before Colonoscopy: A Meta-Analysis of Randomized Controlled Trials

**DOI:** 10.7759/cureus.96705

**Published:** 2025-11-12

**Authors:** Ni Yan, Lingxia Wu, Ji Wang, Lixia Huang, Lefang Wu, Guoyong Zhou

**Affiliations:** 1 Gastroenterology, Hanyang Hospital Affiliated to Wuhan University of Science and Technology, Wuhan, CHN; 2 Translational Medicine, Translational Medicine Institute, Wuhan College of Arts and Sciences, Wuhan, CHN; 3 Tianyuan Translational Medicine Research and Development Team, School of Medicine, Jianghan University, Wuhan, CHN; 4 Gastroenterology, Xianning First People's Hospital, Xianning, CHN; 5 Pharmacy, Jianghan University, Wuhan, CHN; 6 Hubei Key Laboratory of Cognitive and Affective Disorders, Jianghan University, Wuhan, CHN; 7 Hubei Provincial Demonstration Center for Experimental Medicine Education, School of Medicine, Jianghan University, Wuhan, CHN

**Keywords:** colonoscopy, education, intestinal preparation, meta-analysis, wechat app

## Abstract

Background: Intestinal preparation is a key step in implementing colonoscopy, and there is an urgent need to further improve the quality and detection efficiency of intestinal preparation. This meta-analysis is used to evaluate whether WeChat-based education can effectively promote the quality of intestinal preparation.

Method: According to the PRISMA and Cochrane guidelines, eight fully randomized controlled trials utilizing the WeChat app for educational purposes were included. The meta-analysis focused on evaluating the quality of intestinal preparation and colonoscopy outcomes. The effect size was calculated using relative risk (RR). Additionally, the Cochrane Risk of Bias Assessment Tool was employed to assess the risk of bias in the included studies.

Result: The overall RR of sufficient intestinal preparation rate was 1.14 (95% CI: 1.04-1.24, p=0.0038), and the overall RR of cecal intubation rate was 1.014 (95% CI: 1.00-1.030, p=0.069), indicating a significant improvement in the quality of intestinal preparation in the WeChat-based education group. The RR of adenoma detection rate (ADR) was 1.56 (95% CI: 1.20-2.02, p=0.0009), and the overall RR of polyp detection rate (PDR) was 1.25 (95% CI: 1.03-1.51, p=0.022), indicating a significant improvement in the colonoscopy detection rate in the WeChat-based education group.

Conclusion: WeChat-based education can significantly improve the quality of intestinal preparation and colonoscopy detection rate. These findings provide evidence for WeChat-based education in improving the quality of intestinal preparation.

## Introduction and background

Colonoscopy is widely recognized as the gold standard for diagnosing colorectal lesions and serves as one of the most crucial screening tools for colorectal diseases. The age-standardized incidence rate (ASIR) of colorectal cancer globally is 19.5 cases per 100,000 individuals, with a higher incidence in men (23.4 cases) than in women (16.2 cases) [1,2]. The efficacy of colonoscopy is significantly influenced by the quality of intestinal preparation [3-5]. However, the effectiveness of WeChat-based intestinal preparation education prior to colonoscopy remains to be elucidated.

High-quality intestinal preparation can significantly improve the detection rate of polyps and adenomas [6]. However, bowel preparation is a complex process that involves medical expertise and requires patient cooperation, including frequent use of laxatives, strict dietary restrictions, and a strong psychological impact, all of which can affect the quality of bowel preparation and patient compliance [7,8]. Therefore, providing sufficient guidance and education to patients is crucial for improving the quality of colonoscopy preparation.

At present, various methods have been adopted in clinical practice to improve the quality of intestinal preparation, including booklets [9], videos [10], phone calls [11], and television. Due to the widespread use of WeChat as an indispensable tool for mobile payments and information acquisition, multiple studies have used WeChat as a carrier to improve the quality of intestinal preparation, providing patients with more convenient and clear guidance and education on intestinal preparation. This article conducts a meta-analysis of randomized controlled trials (RCTs) to evaluate the impact of WeChat usage on the quality of bowel preparation in patients undergoing colonoscopy testing.

## Review

Methodology

Registration

This study has been registered with PROSPERO (CRD420250653508).

Inclusion Criteria and Exclusion Criteria

The included studies are RCTs on colonoscopy for detecting intestinal preparation; the intervention group used WeChat for education, while the control group used conventional education methods (distributing paper manuals, booklets, watching videos, or discussing colonoscopy examinations during appointments). The subjects are adult patients aged 18 and above, and the literature is in English. Cohort studies, case-control trials, non-RCTs, and meeting summaries were excluded. Because RCTs are the gold standard for evaluating the effectiveness of intervention measures, we chose them as one of the inclusion criteria.

Retrieval Strategy and Literature Screening

Two researchers independently searched online English databases, including PubMed, Web of Science, Cochrane Library, Embase, and Google Scholar, until March 31, 2025. The search terms are “Bowel Preparation” OR “Cathatic“ OR “Bowel Evacuant“ OR “Evacuant, Bowel“ OR “Purge“ OR “Bowel Evacuants“ OR “Evacuants, Bowel“ OR “Purges” OR “Bowel Preparation Solutions, Bowel” OR “Solutions, Bowel Preparation” OR “Bowel Preparation Solution, Bowel” OR “Solution, Bowel Preparation” and (“WeChat” OR “Weixin”) and (“Random” OR “Randomized”).

Data Collection and Analysis

Based on the bias risk assessment tool provided by the Cochrane Collaboration, we formed two independent groups to evaluate the bias of the included literature. If differences persisted, a third researcher was consulted to discuss and reach a consensus. By reading the titles and abstracts, we excluded studies that did not meet the criteria, then downloaded the full texts to exclude studies without complete data. Finally, eight studies were included in our meta-analysis [12-19].

Data Extraction

Extracted the following relevant data from each study: author, publication year, sample size, mean patient age, gender distribution, education method employed, intestinal preparation quality assessment scale, and other pertinent information. The primary outcomes of interest were adenoma detection rate, polyp detection rate, adequate bowel preparation, and cecal intubation rate. During data extraction, the included studies were primarily evaluated by two researchers. In the event of disagreement regarding the inclusion of a specific study, a third researcher was consulted to make a final determination based on study quality and predefined criteria. The quality indicators of intestinal preparation were assessed and confirmed by two practicing nursing physicians.

Data Analysis Methods

We used R (version 4.4.0) for meta-analysis of data extracted from RCTs. For continuous data, we input the mean and standard deviation. For the comparison of rates, we used relative risk (RR) and 95% CI. To compare the degree of variation between the population and subgroups, we calculated the heterogeneity assessment value (I2). I2 heterogeneity between 0% and 40% may not be significant; 30% to 60% represents moderate heterogeneity; 50% to 90% represents substantial heterogeneity; 75% to 100% represents significant heterogeneity (Cochrane Handbook for Systematic Reviews of Interventions). We used the Q-statistic (chi2) to evaluate the significance of I2, where a P-value less than 0.05 indicates heterogeneity. When I2 was greater than 50%, the random effects model was chosen, and when I2 was less than 50%, the fixed effects model was used. In cases of significant heterogeneity, the leave-one-out method was applied to evaluate the source of heterogeneity. The Egger test was used to evaluate publication bias.

Publication Bias

**Figure 1:**
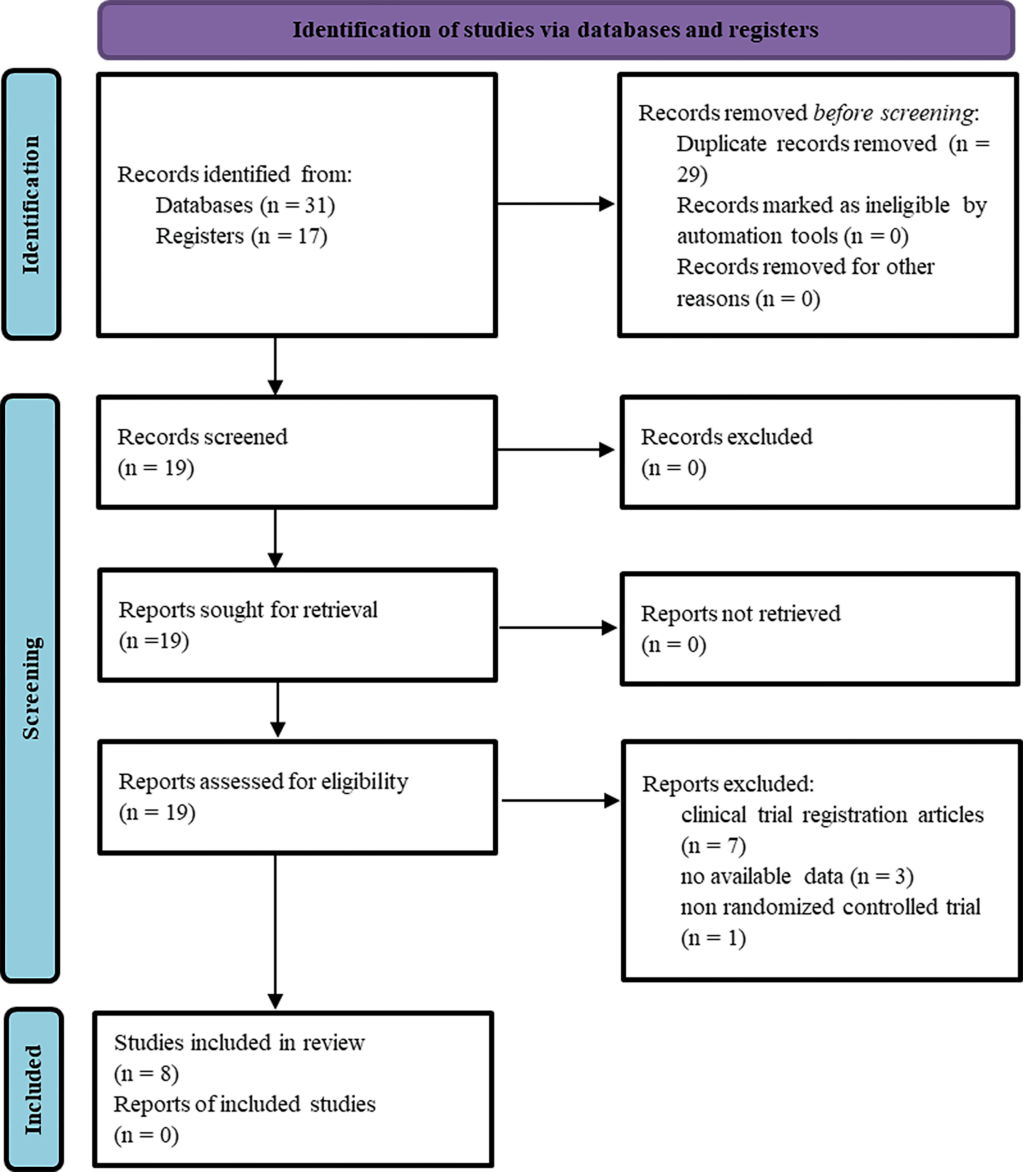
PRISMA flowchart PRISMA, Preferred Reporting Items for Systematic Reviews and Meta-Analyses

The quality of the included studies was assessed using the Cochrane Risk of Bias Tool [20]. To reduce bias, two reviewers independently evaluated the studies based on inclusion and exclusion criteria by reading titles and abstracts. In the event of divergent opinions, a third reviewer was consulted to reach a final decision. All included studies underwent a comprehensive review by three reviewers and were ultimately incorporated into this study. The entire review process adhered to the PRISMA (Preferred Reporting Items for Systematic Reviews and Meta-Analyses) guidelines, as depicted in Figure 1.

Results

Inclusion of Studies and Their Characteristics

A comprehensive search across multiple databases was conducted to identify relevant studies. Specifically, the PubMed database retrieved eight articles, EMBase yielded seven articles, the Cochrane Library identified 16 articles, Web of Science retrieved six articles, and Google Scholar retrieved 11 articles. In total, this initial search phase resulted in 48 articles. After rigorously screening for duplicates, 29 were removed. Additionally, seven clinical trial registration files and four articles that did not meet the predetermined inclusion criteria were excluded. Following this meticulous selection process, eight articles were ultimately retained for the meta-analysis (Figure 1). The detailed characteristics of these included studies are presented in Table 1, which encompasses the overall sample size, research protocol, and other pertinent information for each study.

**Table 1:** Study characteristics ADR, adenoma detection rate; PDR, polyp detection rate; E, experiment number; C, control number

Studies included	Xiaoyu Kang et al., 2015 [12]	Fen Xu et al., 2020 [13]	Xia Li et al., 2023 [14]	Shu-Ling Wang et al., 2018 [15]	Xiaxia Zhao et al., 2023 [16]	Jing Wen et al., 2022 [17]	Qing-xia Zhang et al., 2018 [18]	Xin Yang et al., 2024 [19]
Total number - E	387	100	284	128	158	478	542	818
Total number - C	383	98	280	127	158	473	534	781
ADR (E, C)	72, 46	75, 65	54, 22	6, 5	146, 89			
PDR (E, C)		90, 85	87, 54	44, 41	192, 150			
Cecal intubation rate (E, C)	343, 328	284, 279	127, 125	152, 155	459, 443	489, 469		
Adequate bowel preparation (E, C)	318, 266		114, 83	125, 97	426, 396	489, 469	754, 687	

Evaluate the Risk of Bias

**Figure 2:**
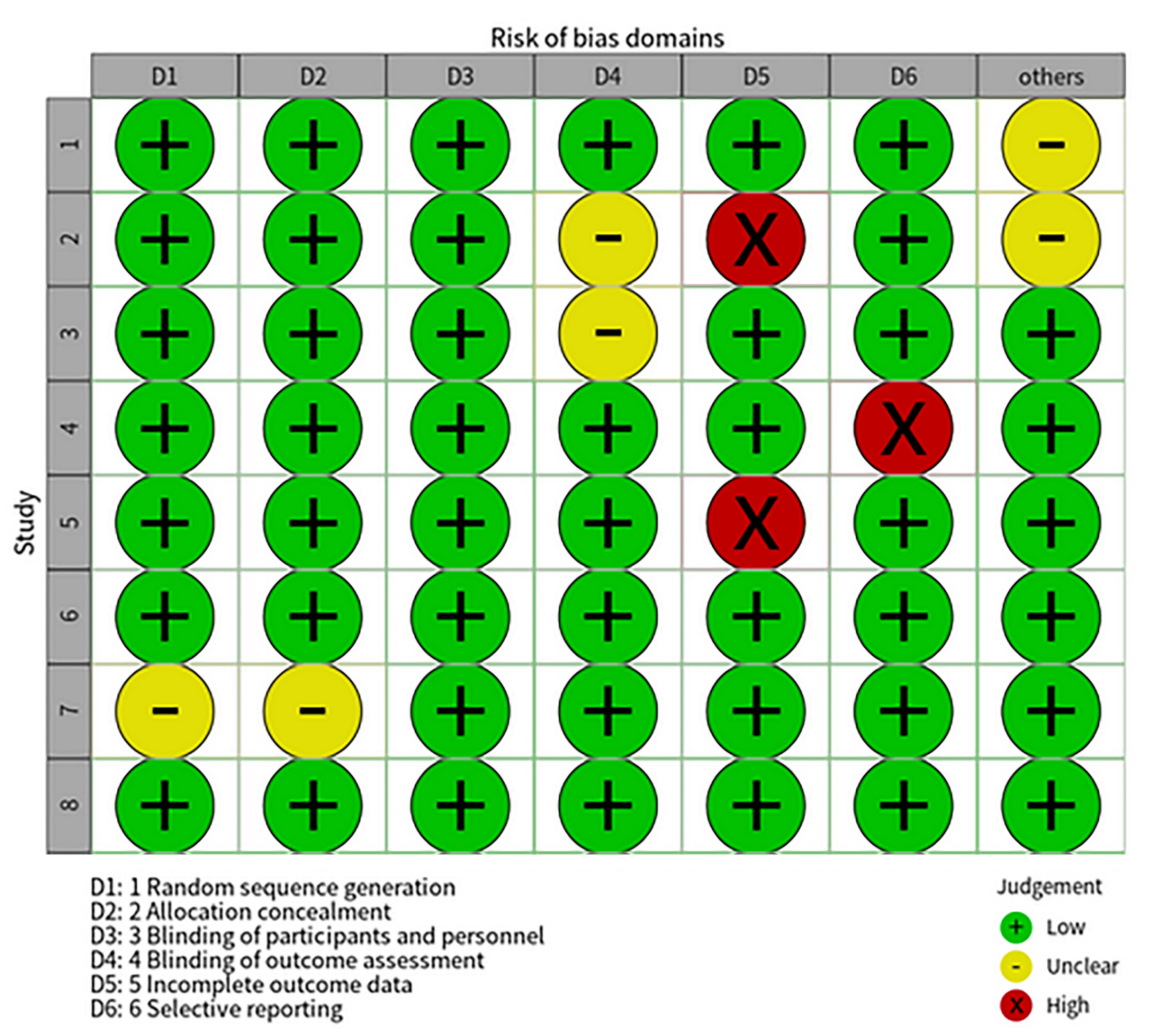
Assessment of risk of bias using the Cochrane Risk of Bias Assessment Tool The included studies were evaluated using the Cochrane Risk of Bias Assessment Tool [20]. Study 1 is Xiaoyu Kang et al. (2015) [12]; Study 2 is Fen Xu et al. (2020) [13]; Study 3 is Xia Li et al. (2023) [14]; Study 4 is Shu Ling Wang et al. (2018) [15]; Study 5 is Xiaxia Zhao et al. (2023) [16]; Study 6 is Jing Wen et al. (2022) [17]; Study 7 is Qing Xia Zhang et al. (2018) [18]; and Study 8 is Xin Yang et al. (2024) [19].

The Cochrane Risk of Bias Assessment Tool was used to conduct risk assessment of the included literature, including random sequence generation, random allocation concealment, blinding of researchers and subjects, blinding evaluation of research results, completeness of outcome data, selective reporting of research results, and other sources of bias. Overall, the risk of bias was relatively low, as shown in Figure 2.

To further clarify the sources of heterogeneity, we summarized the possible sources of heterogeneity in the included studies (Table 2), including the methods used, age, publication year, intervention measures, and evaluation methods. As seen in Kang et al. [12], Ottawa score criteria were used, while other studies used the Boston Bowel Preparation Scale (BBPS) scoring criteria. There is a smaller age range in Fen Xu's research [13], which may have an impact on the adenoma detection rate (ADR) and other factors. For publication bias, the funnel plot and Egger test methods were both used for testing. The p-values of the Egger test for ADR, polyp detection rate (PDR), cecal intubation rate, and adequate bowel preparation were found to be 0.85, 0.70, 0.26, and 0.01, respectively. Correspondingly, the distribution of funnel plots also indicates that adequate bowel preparation has publication bias, while other items do not. Our results indicate that, except for publication bias in intestinal preparation rate, there is no publication bias in other indicators.

**Table 2:** Possible sources of heterogeneity in the included studies MC, multicenter; SC, single-center; RCT, randomized controlled trial; BBPS, Boston Bowel Preparation Scale

Items	Xiaoyu Kang et al., 2015 [12]	Fen Xu et al., 2020 [13]	Xia Li et al., 2023 [14]	Shu-Ling Wang et al., 2018 [15]	Xiaxia Zhao et al., 2023 [16]	Jing Wen et al., 2022 [17]	Qing-xia Zhang et al., 2018 [18]	Xin Yang et al., 2024 [19]
Research method	MC, prospective study, RCT	SC, prospective study, RCT	MC, RCT	SC, prospective, endoscopist blinded, RCT	MC, prospective, RCT	MC, prospective, endoscopist blinded, RCT	SC, prospective study, RCT	MC, prospective study, RCT
Age	18-80	35-70	18-80	18-80	18-80	18-75	18-75	18 years and above
Year	2014	2020	2020	2016	2022	2020	2017	2015
Processing method	Standard education + WeChat interactive information transmission	WeChat video education	WeChat video education	WeChat and SMS reminders	WeChat strengthens education and automatically reminds	WeChat application-assisted education	WeChat public account	WeChat enhanced education
Evaluation method	Ottawa score	BBPS score	None	BBPS score	BBPS score	BBPS score	Aronchick scale, BBPS score	BBPS score

Quality of Intestinal Preparation

**Figure 3:**
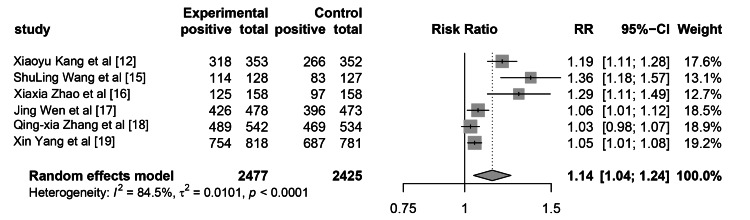
The impact of WeChat-based education on intestinal preparation quality

There were 2477 patients in the WeChat-based education group, of whom 2226 had sufficient intestinal preparation, resulting in a sufficient intestinal preparation rate of 90.0%. There were 2425 patients in the control group, of whom 1998 had sufficient intestinal preparation, with a sufficient intestinal preparation rate of 82.4%. The heterogeneity test showed that I2 was 84.5%, with a P-value less than 0.0001, indicating significant heterogeneity. The RR for the summary of sufficient intestinal preparation rate was 1.14 (95% CI: 1.04-1.24, p=0.0038), as shown in Figure 3.

**Figure 4:**
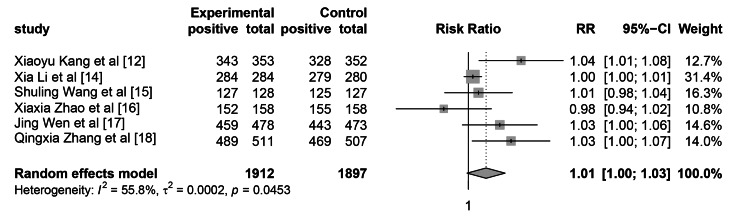
The impact of WeChat-based education on cecal intubation rate

There were a total of 1912 patients in the WeChat-based education group, of whom 1854 cases achieved successful cecal intubation, resulting in a success rate of 97.3%. In the control group, 1897 patients were included, with 1799 successful cecal intubations, yielding a cecal intubation rate of 94.8%. The heterogeneity test showed that I2 was 55.8%, with a P-value less than 0.045, indicating significant heterogeneity. The summary RR for cecal intubation rate was 1.014 (95% CI: 1.00-1.030, p=0.069), as shown in Figure 4.

Colonoscopy Results

**Figure 5:**
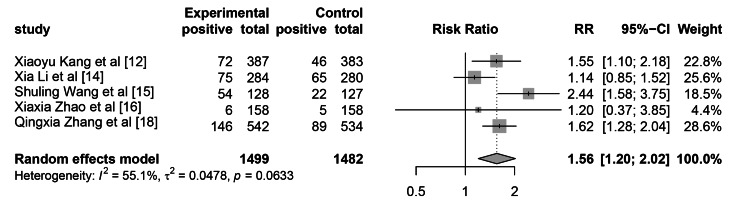
The impact of WeChat-based education on ADR ADR, adenoma detection rate

There were 1499 patients in the WeChat-based education group and 1482 patients in the control group who underwent colonoscopy for adenomas. The heterogeneity test showed that I2 was 55.1%, with a P-value of 0.063. The RR for ADR was 1.56 (95% CI: 1.20-2.02, p=0.0009) as shown in Figure 5. Our results indicate that using the WeChat app for education can significantly improve ADR.

The leave-one-out analysis showed that the results of Xiao Li et al. [14] were abnormal. This may be related to the education method, as this study used only video education, while several other studies provided more diverse educational approaches.

**Figure 6:**
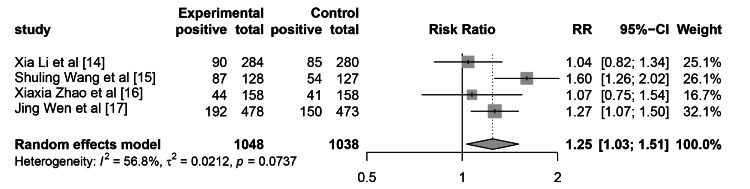
The impact of WeChat-based education on PDR PDR, polyp detection rate

There were 1048 patients in the WeChat-based education group and 1038 patients in the control group who underwent colonoscopy for polyps. The total RR of PDR is 1.25 (95% CI: 1.03-1.51, p=0.022), as shown in Figure 6. The results indicate that WeChat-based education can significantly improve the PDR. The heterogeneity test results showed that I2 was 56.8%, with a P-value of 0.074, indicating no significant heterogeneity. Use of the leave-one-out method reveals that heterogeneity mainly comes from Shu-Ling Wang et al. [15]. Calculation showed that the PDR in this study was significantly higher than in other groups, including both the WeChat group and the control group.

Discussion

This study consisted of eight RCTs. The meta-analysis results showed that the intestinal preparation rate of patients in the WeChat-based education group was significantly higher than that of the control group, the BBPS score was significantly higher than that of the control group, and the cecal intubation rate was relatively higher than that of the control group. The quality of intestinal preparation and colonoscopy results in the WeChat-based education group was higher than that in the control group.

The accuracy and safety of colonoscopy diagnosis largely depend on the quality of intestinal preparation, which includes not only the standardized use of laxatives but also strict dietary control. The entire process is complex and difficult for non-medical professionals to understand, let alone strictly follow. Therefore, numerous studies have shown that strengthening intestinal preparation education can significantly improve the quality of intestinal preparation [3,8,21-23].

With its extensive user base and multifunctionality, the WeChat app has shown great potential in intestinal preparation education. WeChat supports the display of multimedia content, and medical staff can create illustrated guides for intestinal preparation, even recording short videos to demonstrate the correct way to take laxatives, specific requirements for dietary restrictions, and deliver them to patients in an intuitive and understandable form [13,24]. Second, WeChat's instant messaging function allows patients to have real-time online consultations and Q&A with medical staff. Patients can receive timely answers to any questions they encounter during the preparation process, thereby improving their understanding and compliance. In addition, through the WeChat official account or applet, researchers can regularly push knowledge about bowel preparation to strengthen patients' cognition [25,26]. This convenient and efficient education method not only enhances patients' understanding of the intestinal preparation process but also strengthens the interaction between doctors and patients, which helps to improve the quality of intestinal preparation and ultimately enhances the effectiveness of colonoscopy examination.

The summary of sufficient intestinal preparation rates found significant heterogeneity among studies. The sensitivity analysis using the leave-one-out method showed no significant change in heterogeneity, indicating the reliability of our results. However, it also suggests that heterogeneity still exists and originates from other sources.

The WeChat app is not merely a social platform but also a software capable of providing customized services, such as the WeChat official account. A randomized experiment utilizing the WeChat official account was conducted [27], and the results indicated that education via the official account was superior to that of the control group. In addition to the WeChat app being employed for intestinal preparation education, WhatsApp, another widely used social media app, has also been utilized for this purpose. Studies have demonstrated that both the quality of intestinal preparation and colonoscopy outcomes have significantly improved [28]. These findings suggest that social media can effectively enhance the quality of intestinal preparation through educational interventions.

Limitations

Of course, our meta-analysis still has some shortcomings. First, although we conducted sensitivity analysis, we were unable to perform stratified analysis due to the limited sample size, resulting in insufficient exploration of the sources of heterogeneity. Second, because of population and regional differences across studies, the education level and proficiency in using the WeChat app vary among participants in each region, inevitably contributing to heterogeneity among studies.

## Conclusions

This meta-analysis of eight randomized controlled trials demonstrates that WeChat-based education significantly enhances the quality of intestinal preparation and colonoscopy detection rates compared with conventional education methods. However, our study also acknowledges certain limitations. Despite the widespread use of the WeChat app, it remains a significant challenge for the elderly or those unwilling to use mobile social media for gut preparation education. The significant heterogeneity observed in some outcomes, such as ADR, indicates the need for cautious interpretation of the results and highlights the importance of further research to explore the sources of heterogeneity. Given that all the included studies originated from Mainland China, further validation is required to confirm the generalizability of these findings to a broader global context.

Despite these limitations, the results provide valuable insights into the application of the WeChat app in gastrointestinal preparation, suggesting that this digital tool could be effectively integrated into clinical practice to enhance patient outcomes in colonoscopy procedures. Future studies with larger sample sizes and more rigorous designs are warranted to confirm these findings and to further explore the optimal methods for implementing WeChat-based education in diverse clinical settings.
